# Synthesis, Structural Characterization, Hirshfeld Surface Analysis, and Evaluation of Nonlinear Optical Properties of Novel Cocrystal of Acridine with 2,4-Dihydroxybenzaldehyde

**DOI:** 10.3390/ma18071492

**Published:** 2025-03-27

**Authors:** Patryk Nowak, Artur Sikorski

**Affiliations:** Faculty of Chemistry, University of Gdańsk, W. Stwosza 63, 80-308 Gdańsk, Poland

**Keywords:** acridine, 2,4-dihydroxybenzaldehyde, cocrystal, crystal structure, nonlinear optic material

## Abstract

A cocrystal of acridine with 2,4-dihydroxybenzaldehyde (2:1 stoichiometric ratio) was synthesized, spectrally and structurally characterized using TG, DSC, ATR-FTIR and Single-Crystal XRD methods and Hirshfeld surface analysis, and its nonlinear optical properties were investigated by DFT at the B3LYP/6-311++Glevel. The obtained compound crystallizes in the noncentrosymmetric *P*2_1_ monoclinic space group, with two molecules of acridine and one molecule of 2,4-dihydroxybenzaldehyde in the asymmetric unit. The strong O_(aldehyde)_–H⋯N_(acridine)_ and weak C_(aldehyde)_–H⋯O_(aldehyde)_ and C_(aldehyde)_–H⋯O_(aldehyde)_ hydrogen bonds, as well as π_(acridine)_–π_(acridine)_ and C_(acridine)_–H⋯π_(aldehyde)_ interactions, are present in the crystal lattice of the title compound. The calculated energy gap (ΔE) between the HOMO-LUMO surfaces shows charge transfer interactions due to the π-π* transitions among the molecules. The calculated first and second hyperpolarizability values indicate that obtained cocrystal is a promising candidate for nonlinear optical applications.

## 1. Introduction

Crystalline solids of organic compounds play a fundamental role in chemical engineering and materials science. Compared to amorphous or semi-crystalline organic semiconducting materials, artificially grown single crystals obtained through slow evaporation methods are considered ideal for fundamental research and technological applications in the optics and electronics industries [1]. Organic cocrystals represent a specific type of crystalline material formed from two or more neutral coformers [2]. In cocrystals, different molecules interact through supramolecular interactions, resulting in a unique molecular arrangement within the crystal lattice. This distinct structure can lead to unpredictable physicochemical properties, including high conductivity [3], nonlinear optics (NLO) [4,5,6,7,8], charge transport [9,10], ferroelectricity [11,12,13], light energy conversion [14,15], and phosphorescence [16], particularly when compared to single-component materials.

Organic π-conjugated materials are widely used in photoelectronic applications due to their π-bond system, which enables charge delocalization and high electron mobility [17]. In NLO, the extent of charge transfer across chromophores influences second-harmonic generation (SHG) output. Functionalizing both ends of the π-system with suitable donor and acceptor groups enhances asymmetric electron distribution in ground and excited states, increasing optical nonlinearity [18]. In the context of multicomponent crystals, the noncentrosymmetric space group is one of the most crucial issues in developing highly successful second-order NLO crystalline materials [19,20]. The cocrystallization technique is widely used to obtain noncentrosymmetric structures, where, in addition to the π-bond system, hydrogen bonds play a crucial role in molecular recognition, and enhances NLO properties due to their strength and directionality [21,22].

As reported by Rajkumar [23] and others [24,25,26], there is the possibility to obtain potentially SHG-active noncentrosymmetric acridine-based cocrystals, whereas Lakshmanaperumal et al. [27] and others [28,29,30] showed that it is also possible to obtain multicomponent crystals of hydroxybenzaldehydes. A search of the Cambridge Structural Database (CSD version 5.43, update March 2025) shows that there are only three crystal structures of cocrystals derived from acridines and benzaldehyde derivatives [31,32].

**Scheme 1:**
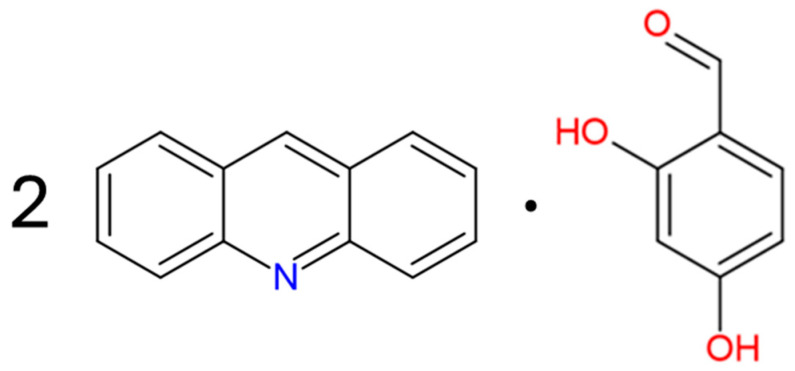
Chemical structure of the bis(acridine)–2,4-dihydroxybenzaldehyde cocrystal.

In view of the above, as a continuation of our previous study concerning multicomponent crystals formed from acridines and benzaldehydes [32], here we describe the synthesis and structural characterization of new noncentrosymmetric cocrystal derived from acridine and 2,4-dihydroxybenzaldehyde (2:1 stoichiometric ratio) (Scheme 1). In particular, we present the results of thermal, spectroscopic, and structural studies, as well as analyses of intermolecular interactions, and the Hirshfeld surface and energetic properties of the cocrystal obtained, accompanied by theoretical NLO calculations.

## 2. Materials and Methods

All reagents were purchased from Sigma-Aldrich (St. Louis, MO, USA). The purities of the reagents and the product were verified by melting point measurements, performed using a Büchi M-545 apparatus. Crystal images were captured using a Discovery Femto Polar Digital Microscope with a 3 MP Digital Camera.

### 2.1. Synthesis of Bis(acridine)–2,4-dihydroxybenzaldehyde Cocrystal

Acridine (0.160 g, 0.894 mmol) and 2,4-hydroxybenzaldehyde (0.062 g, 0.449 mmol) were dissolved in 10 mL of methanol/dichloromethane (1:1, *v*:*v*) mixture. The mixture was refluxed via continuous magnetic stirring for 30 min. The resulting solution was left to evaporate at room temperature for several days, yielding a yellow powder. The obtained product was filtered and subsequently recrystallized in the same mixture. The solution was then left to evaporate at 4 °C for a few days, yielding dark yellow crystals of bis(acridine)–2,4-dihydroxybenzaldehyde (Figure S1). The characterization data are provided below. Yield: >90%; m.p.: 141.0 °C. Element. analysis calc./found for C_33_H_24_N_2_O_3_: C 79.82/79.67, H 4.87/4.85, N 5.64/5.57.

### 2.2. Thermogravimetry (TG) and Differential Scanning Calorimetry (DSC)

The thermal analysis of char samples was performed using simultaneous TG-DSC analyser Netzsch STA 449 F3 Jupiter (NETZSCH Analyzing & Testing, Bavaria, Germany). The sample (3 mg) was heated with a heating rate of 10 °C/min from 30 to 1000 °C in a N_2_ atmosphere.

### 2.3. Attenuated Total Reflectance-Fourier Transform Infrared Spectroscopy (ATR-FTIR)

The FTIR spectrum was acquired using a Perkin Elmer Spectrum 3™ instrument (Perkin Elmer, Waltham, MA, USA) equipped with an attenuated total reflectance (ATR) accessory. Measurements were performed at room temperature in reflective mode, with a spectral range of 4000 to 500 cm^−1^, a resolution of 4 cm^−1^, and 16 scans averaged for each measurement. The resulting FTIR spectrum was processed and baseline-corrected using PerkinElmer Spectrum IR software (version 10.7.2).

### 2.4. X-ray Measurements and Refinements

A single-crystal X-ray diffraction experiment was performed at T = 291(2) K (Table 1) using an Oxford Diffraction Gemini R ULTRA Ruby CCD diffractometer (Oxford Diffraction, Oxford, UK) (λ_Cu_ = 1.54184 Å) [33]. The structure was solved and refined using the SHELX package programs (ver. 2017/1) [34,35]. H atoms from hydroxyl groups were located on a difference Fourier map and refined freely, whereas H atoms bound to C atoms were placed geometrically and refined using a riding model with C–H = 0.93 Å and U_iso_(H) = 1.2 U_eq_(C). The following software was used to analyze the results and prepare the figures: PLATON [36], ORTEPII [37] and Mercury [38].

### 2.5. Hirshfeld Surface, 2D Fingerprint Plots, and Energy Framework

Ab initio calculations of the Hirshfeld surfaces, fingerprint plots, and energy frameworks were performed using the CrystalExplorer program (ver. 21.5) [39,40]. The wave functions of each molecule and the pairwise interactions for the energy framework estimation were calculated using Tonto [41], with the B3LYP DFT method and the 6–31 G(d,p) basis set, as implemented in CrystalExplorer. Interaction energy calculations between each molecule and its chemical neighborhood were performed, generating a cluster within a 3.8 Å radius. The selected energies in specific directions are depicted as energy frameworks, which appear as colored cylinders connecting the centroids of interacting molecular pairs. For visual clarity, a tube size scale of 50 was used for energy framework representation, and the cut-off value for energies was set at 9 kJ/mol.

### 2.6. Theoretical Calculations

Quantum chemical calculations of electrostatic potential (MEP) and frontier molecular orbital (FMO) were carried out using the Gaussian 09W software [42]. All calculations were performed by DFT/B3LYP quantum chemical function with a triple zeta 6–311++G basis set and visualized in the GaussView software (ver. 5) [43]. An analysis of NLO data was performed using the Multiwfn software (ver. 3.7) [44,45].

## 3. Results and Discussion

### 3.1. Thermal Analysis

**Figure 1:**
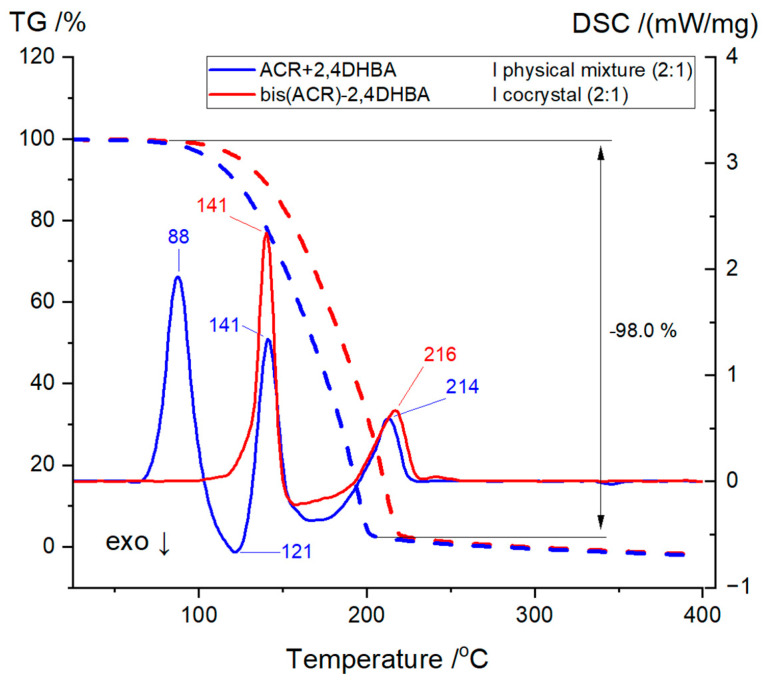
TG curve (dashed) and DSC curve (solid) of the bis(acridine)–2,4-dihydroxybenzaldehyde (red line) compared to the corresponding physical mixture of acridine and 2,4-dihydroxybenzaldehyde (blue line).

To provide evidence for the formation of a new crystalline cocrystal and to further investigate its thermal behavior and stability, TG/DSC were used to measure thermal effects of both the obtained bis(acridine)–2,4-dihydroxybenzaldehyde cocrystal (bis(ACR)–2,4DHBA) and its physical mixture (ACR + 2,4DHBA) in the same molar ratio (2:1) (Figure 1). For the cocrystal sample, a single-phase transition peak appears at 141 °C during heating, corresponding to the melting point of the cocrystal. The second peak at 216 °C corresponds to a decomposition process, resulting in a 98% mass loss. This pattern is similar to one we observed in our recent studies on two cocrystals derived from acridine and 3- and 4-hydroxybenzaldehydes [32]. In contrast, during the thermal analysis of the physical mixture, we observed a partial melting process associated with the melting of acridine (single melting peak at 88 °C), followed by the recrystallization of the molten fraction. This suggests a reaction between acridine and 2,4-dihydroxybenzaldehyde, as indicated by an exothermic peak at 121 °C, and subsequent melting at 141 °C, confirming the possibility of cocrystal formation upon heating.

### 3.2. ATR-FTIR Analysis

**Figure 2:**
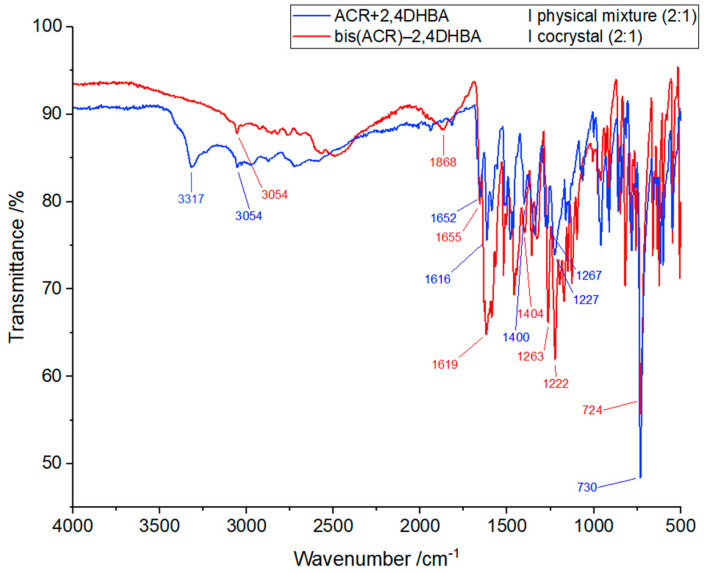
ATR-FTIR spectrum of bis(acridine)–2,4-dihydroxybenzaldehyde (red line) compared to the corresponding physical mixture of acridine and 2,4-dihydroxybenzaldehyde (blue line).

The ATR-FTIR spectra were used to analyze changes between the cocrystal of acridine with 2,4-dihydroxybenzaldehyde and its physical mixture in the same molar ratio (2:1) (Figure 2). The identified functional groups and their corresponding peaks are listed in Table S1. The spectrum of the physical mixture shows characteristic bands at 3317 cm^−1^, 3054 cm^−1^, 1652 cm^−1^, 1616–1400 cm^−1^, 1267–1227 cm^−1^, and 730 cm^−1^, corresponding to O–H stretching vibrations, aromatic C–H stretching vibrations, C=O stretching vibrations, C=C and C=N skeletal stretching vibrations, C–O phenolic stretching vibrations, and C–H out-of-plane bending vibrations, respectively. For the cocrystal, characteristic bands are observed at 3054 cm^−1^, 1655 cm^−1^, 1619–1404 cm^−1^, 1263–1222 cm^−1^, and 724 cm^−1^, corresponding to aromatic C–H stretching vibrations, C=O stretching vibrations, C=C and C=N skeletal stretching vibrations, C–O phenolic stretching vibrations and C–H out-of-plane bending vibrations, respectively [46]. In turn, the band associated with the stretching vibrations of the hydroxyl group (A-type) in 2,4-dihydroxybenzaldehyde is no longer present. A similar pattern was observed in the case of acridine-3-hydroxybenzaldehyde and acridine-4-hydroxybenzaldehyde, as reported in our recent studies [32]. Interestingly, a new band appears at 1868 cm^−1^, which, to the best of our knowledge, can be regarded as a strong N_(acridine)_···H–O_(2,4-dihydroxybenzaldehyde)_ low-barrier hydrogen bond [47]. The bands in the 2500–3000 cm^−1^ range can be attributed to C–H stretching, as seen in other acridine complexes, and Fermi resonance between stretching and bending in the plane of hydrogen-bonded (B-type) O–H groups, and these bands are usually determined for complexes with strong hydrogen bonds [48,49].

### 3.3. Crystal Structure and Intermolecular Interactions

**Figure 3:**
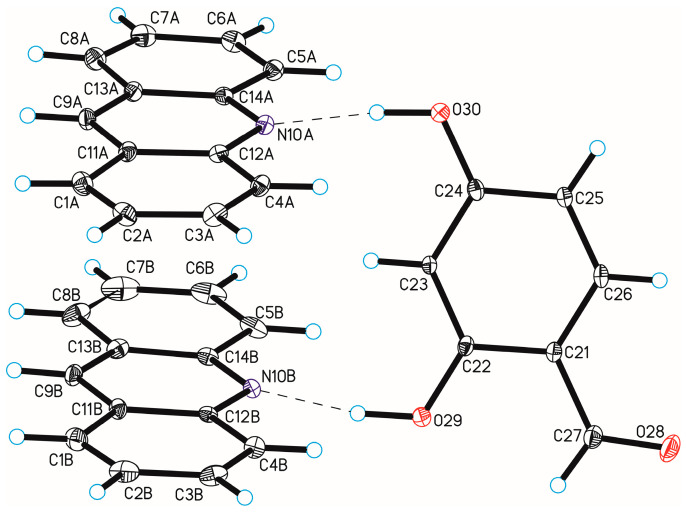
Molecular structure of bis(ACR)–2,4DHBA cocrystal showing the atom-labeling scheme (displacement ellipsoids are drawn at the 25% probability level; hydrogen bonds are represented by dashed line).

The SCXRD experiment shows that the obtained compound crystallizes as cocrystal in the noncentrosymmetric *P*2_1_ monoclinic space group, with two molecules of acridine and one molecule of 2,4-dihydroxybenzaldehyde in the asymmetric unit (Figure 3 and Table 1). The title compound is not isostructural with the recently studied cocrystals acridine–3-hydroxybenzaldehyde (triclinic *P*-1) and acridine–4-hydroxybenzaldehyde, which crystallize in the monoclinic *P*2_1_/c space group [32]. The C–O bond lengths in the hydroxyl groups of the benzaldehyde molecule are 1.32 Å, while the C12–N10–C14 valence angles in the acridine molecules are 119.7° and 118.7°, which confirm that proton transfer does not occur between the hydroxyl groups of benzaldehyde and the endocyclic N-atom of acridines [31,50]. In the cocrystals of acridine with 3-hydroxybenzaldehyde and 4-hydroxybenzaldehyde, these values are similar (1.36 Å and 119.1°, 1.35 Å and 118.8°, respectively). As we previously showed [50], the C12–N10–C14 angle ranges of 118° to 120° are characteristic of acridine cocrystals, whereas they range from 123° to 124° for acridinium salts.

**Figure 4:**
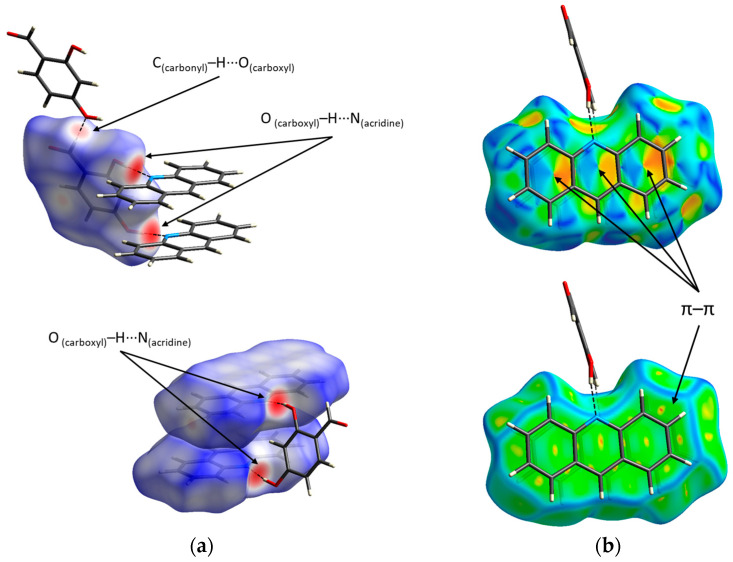
Hirshfeld surface of the bis(ACR)–2,4DHBA cocrystal, mapped over (**a**) *d*_norm_ and (**b**) shape index and curvedness, with hydrogen bonds shown as black dashed lines. For d*_norm_*: Red indicates close contacts and blue represents long-range contacts. For the shape index: Orange shows concave areas (hydrogen bond acceptors or π interactions) and blue indicates convex areas (hydrogen bond donors or π-electron density). For curvedness: Green corresponds to flat regions and blue indicates highly curved areas. The Rescale Surface Property ranges from −0.7 to 1.2 arbitrary units for *d*_norm_, from −1.0 to 1.0 arbitrary units for shape index and from −4.0 to 0.4 for curvedness.

In the cocrystal of bis(ACR)–2,4DHBA, the 2,4-dihydroxybenzaldehyde molecule interacts with the acridine molecules through the O_(carboxyl)_–H···N_(acridine)_ hydrogen bond [*d*(H29···N10B) = 1.70(12) Å and <(O29–H29···N10B) = 167(13)°, and *d*(H30···N10A) = 1.75(7) Å and <(O30–H30···N10A) = 171(4)°] to form a noncentrosymmetric, cyclic trimer (Table S2). Hirshfeld surfaces mapped over *d*_norm_ for individual mers are shown in Figure 4a. This trimer also features π–π stacking formed between two acridine moieties with a centroid···centroid distance [*d*(Cg···Cg)] ranging from 3.789 to 4.043 Å (Table S3). The Hirshfeld surface mapped with the shape index function shows the presence of ‘bow-tie’ patterns, indicating aromatic π–π stacking interactions, as depicted at Figure 4b. Additionally, the large green flat region between molecules provides further evidence of aromatic stacking interactions. These findings are consistent with results reported by Gumus et al. [51] and McKinnon et al. [52].

**Figure 5:**
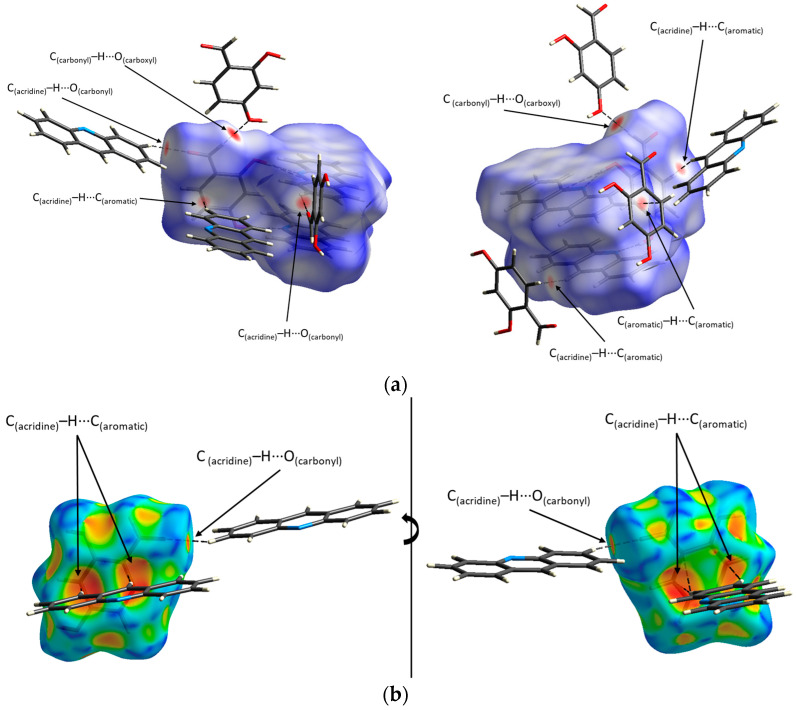
Hirshfeld surface for bis(ACR)–2,4DHBA molecules mapped over (**a**) *d*_norm_ and (**b**) shape index, with hydrogen bonds shown as black dashed lines.

The analysis of interactions between neighboring trimers reveals the presence of C_(carbonyl)_–H···O_(carboxyl)_ and C_(acridine)_–H···O_(carbonyl)_, hydrogen bonds (Figure 5a, Table S2), along with additional π_(acridine)_–π_(acridine)_ interactions (Table S3). The Hirshfield surface of 2,4-dihydroxybenzaldehyde generated over the shape index also shows the presence of C_(acridine)_–H···π_(aldehyde)_ and C_(aldehyde)_–H··· π_(aldehyde)_ interactions (Figure 5b, Table S4). These interactions contribute to the formation of a three-dimensional network by establishing two main structural motifs.

**Figure 6:**
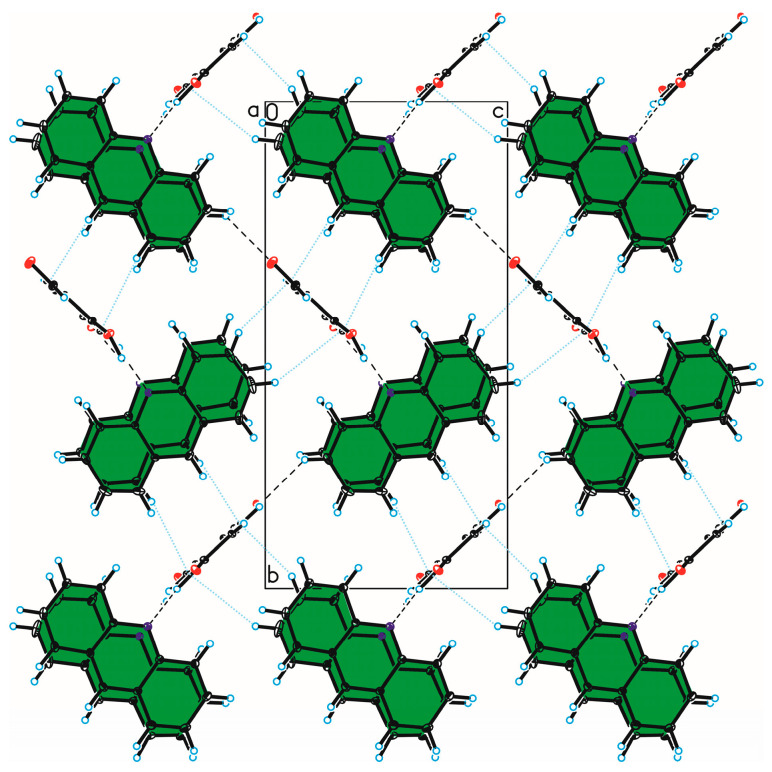
Structural motif of hydrogen-bonded zig-zag chains in the crystal of bis(ACR)–2,4DHBA. Hydrogen bonds are represented by black dashed lines, whereas C–H···π interactions are represented by blue dotted lines.

Adjacent trimers are linked by C_(acridine)_–H···O_(carbonyl)_ hydrogen bonds to form a zig-zag motif along the crystallographic [0 1 0] direction (Figure 6).

**Figure 7:**
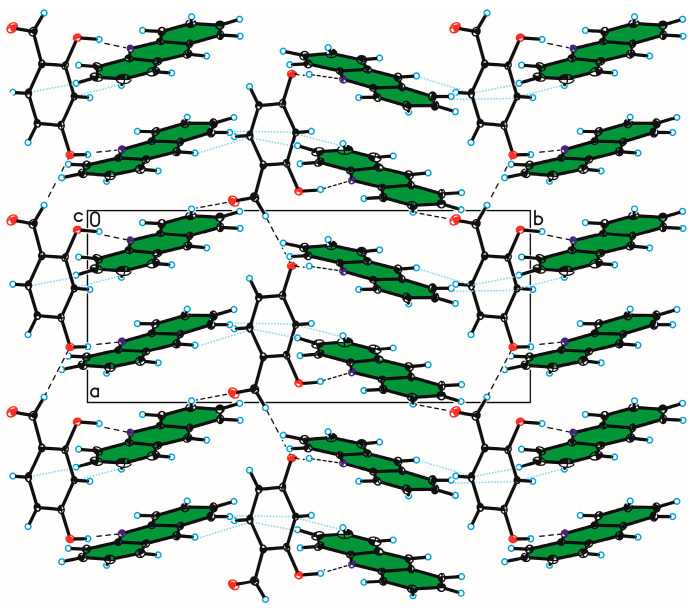
Antiparallel columnar motif in the crystal of bis(ACR)–2,4DHBA, with hydrogen bonds (black dashed lines) and C–H···π interactions (blue dotted lines).

Additionally, π–π interactions, with *d*(Cg···Cg) distances ranging from 3.695 to 3.917 Å, occur between the aromatic rings of acridine moieties, linking independent trimers. As a result, structural motifs of antiparallel columns of π–π stacked head-to-head acridine and 2,4-dihydroxybenzaldehyde molecules are formed along the crystallographic [1 0 0] direction (Figure 7).

**Figure 8:**
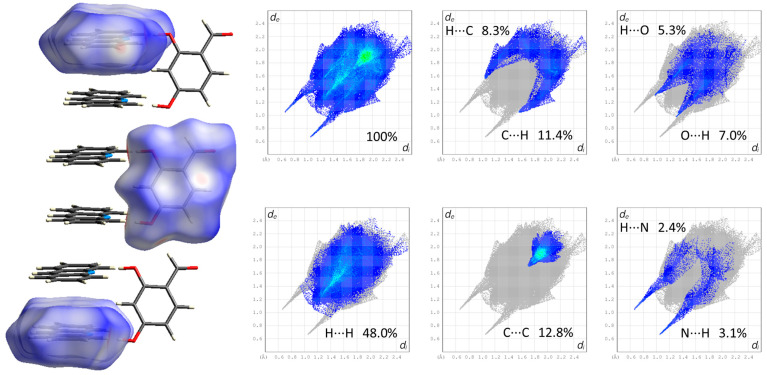
Fingerprint plots illustrating the percentage contribution of the most prominent interactions for the bis(ACR)–2,4DHBA cocrystal.

The analysis of the decomposed 2D fingerprint plots highlights the intermolecular patterns in the cocrystal of bis(ACR)–2,4DHBA [53]. The most prominent interactions are van der Waals forces (H···H contacts), which correspond to 48.0% of the total Hirshfeld surface. The C···H/H···C interactions contribute 19.7%, while the C···C interactions, involved in stacking interactions, account for 12.8%. The O···H/H···O and N···H/H···N intermolecular interactions correspond to C–H···O and O–H···N hydrogen bonds, representing the closest contacts in the structures and appearing as red spots on the *d*_norm_ surface. These interactions are visible as sharp spikes in the fingerprint plots, contributing 12.3% and 5.5% to the total Hirshfeld surface. Interactions with a contribution below 5% were omitted. All 2D fingerprint plots are shown together in Figure 8.

**Figure 9:**
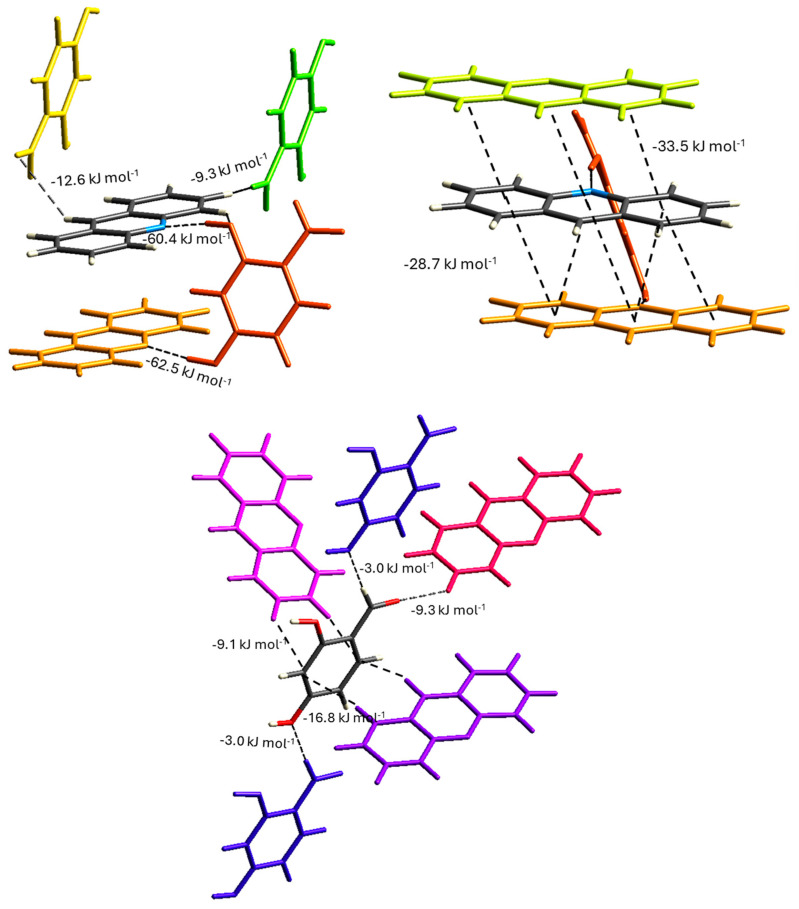
Energy representation of molecular interactions in the bis(ACR)–2,4DHBA cocrystal, showing the central analyzed molecule (non-colored) and surrounding molecules (colored).

The total interaction energies between molecules indicate that the O_(carboxyl)_–H···N_(acridine)_ interactions are the strongest in the crystal lattice, with values of −60.4 kJ/mol for O29–H29···N10B and −62.5 kJ/mol for O30–H30···N10A. The interactions between acridine molecules involved in stacking interactions (−28.7 kJ/mol for stacking within a trimer and −33.5 kJ/mol for stacking between trimers) are the most prominent for the above-mentioned antiparallel columnar motif. These total energy differences between acridine rings may result from the asymmetry in π electron cloud distribution, leading to uneven charge delocalization and further explaining the noncentrosymmetric nature of the cocrystal [54,55]. In contrast, for the zig-zag motif, the total energy of the C3B–H3B···O28 hydrogen bond is relatively low (−9.3 kJ/mol) compared to that of the antiparallel columnar motif, suggesting that the motif is the dominant structural feature. Selected energy interaction values between molecular pairs are presented in Figure 9 and summarized in Figure S2.

**Figure 10:**
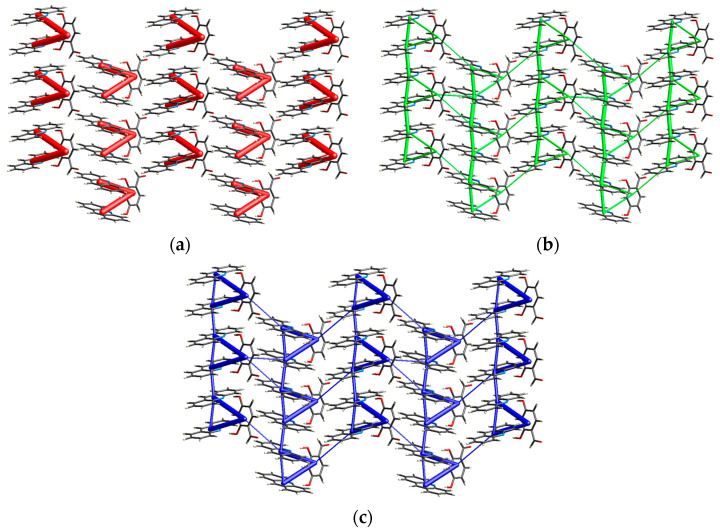
Energy frameworks for a molecular cluster of the bis(ACR)–2,4DHBA cocrystal, depicting electrostatic energy (**a**), dispersion energy (**b**), and total energy (**c**). The tube size is set to 50, with a cut-off of 9 kJ/mol.

Overall, the crystal structure can be classified as a columnar type, as illustrated by the energy framework in Figure 10c. Notably, significantly stronger interactions occur along the antiparallel columnar motif in the [1 0 0] direction compared to the hydrogen bond zig-zag motif in the [0 1 0] direction, with a higher proportion of dispersion contribution (Figure 10a,b).

### 3.4. Theoretical Studies

**Figure 11:**
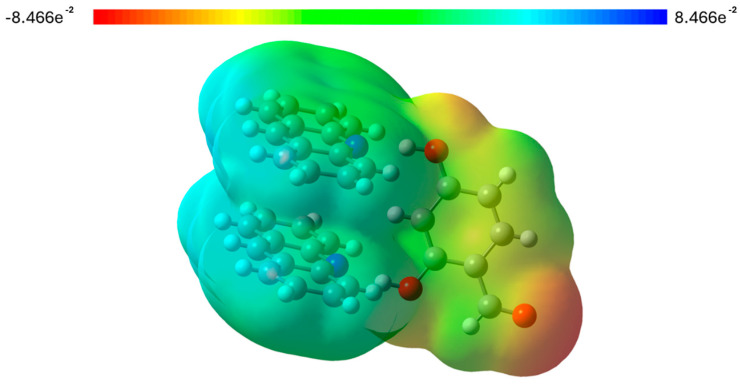
Molecular electrostatic potential (MEP) map of bis(ACR)–2,4DHBA cocrystals with corresponding isovalue contours. The color coding runs from −8.466 a.u (red) to 8.466 a.u (blue).

MEP calculations are a valuable tool for identifying molecular sites involved in electrophilic and nucleophilic interactions, as well as for studying molecular reactivity and the relationship between structure and reactivity [56,57]. The MEP surface mapping of the title cocrystal reveals that the regions of negative electrostatic potential are primarily concentrated around the oxygen atoms, which act as acceptor sites in the formation of C_(carbonyl)_–H···O_(carboxyl)_ and C_(acridine)_–H···O_(carbonyl)_ hydrogen bonds (Figure 11). Similarly, the positive electrostatic potential regions are concentrated over the acridine H atoms, which indicates the formation of the C_(acridine)_–H···O_(carbonyl)_ hydrogen bond, weak C_(acridine)_–H···C_(aromatic)_ and C_(aromatic)_–H···C_(aromatic)_, and van der Waals interactions.

**Figure 12:**
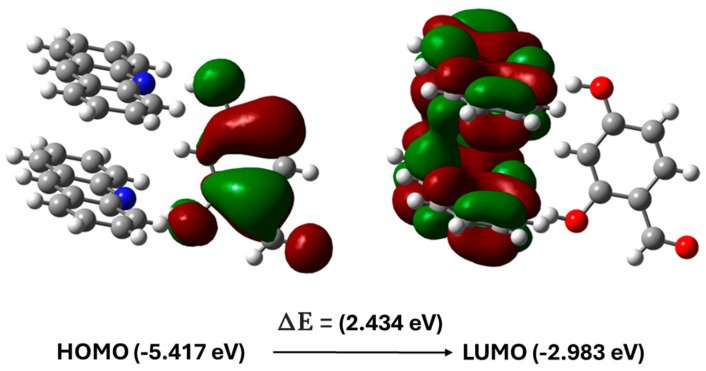
Spatial HOMO-LUMO plots and associated energy gap (ΔE) for bis(ACR)–2,4DHBA cocrystal molecules.

The frontier molecular orbitals show the path of charge transfer in the system and play an important role in determining the electric and optical properties. The highest occupied molecular orbital (HOMO) is typically viewed as nucleophilic, capable of donating electrons, while the lowest unoccupied molecular orbital (LUMO) acts as an electrophilic site, accepting electrons. The HOMO-LUMO energy gap explains the concluding charge transfer interaction within the molecule and is useful in determining molecular electrical transport properties [58,59]. A high HOMO-LUMO energy gap indicates the low chemical reactivity and high kinetic stability of the molecular components based on their electronic transition sites [60]. The HOMO of cocrystal molecules is concentrated over the donor as 2,4-dihydroxyebnzaldehyde, and the LUMO is concentrated over the acceptor acridine, indicating charge transfer from the HOMO of the donor to the unoccupied orbitals LUMO of the acceptor molecules (Figure 12). The calculated energy gap (ΔE) between the HOMO-LUMO surfaces is 2.434 eV, indicating charge transfer interactions due to the π-π* transitions among the molecules. The low ΔE value suggests that the obtained cocrystal exhibits low chemical reactivity and high kinetic stability. This can be related to the absence of strong hydrogen bonds in the zig-zag motif within the crystal lattice. As previously described, the strongest hydrogen bond observed between trimers is C_(carbonyl)_–H···O_(carboxyl)_, with an interaction energy of −9.3 kJ/mol. Similar observations were reported by Rajamoni et al., who studied a series of acridine-based cocrystals [61]. The calculated energy values and corresponding global descriptors are listed in Table S5.

Organic molecules with extended π-conjugation, like our obtained cocrystal, can exhibit enhanced nonlinear optical (NLO) properties [62,63]. To assess charge delocalization and evaluate the nonlinear optical effects in the compound, it is necessary to calculate the first hyperpolarizability and its related components, as these are essential for modulating various optical processes [64,65]. The x, y, and z components of the dipole moment (μ), polarizability (α), and first hyperpolarizability (β) were derived from the Gaussian output file and are given in Table S6.

The calculated mean polarizability and anisotropy of the polarizability of the studied compound were determined to be 5.6119 × 10^−23^ and 3.1282 × 10^−23^ esu, respectively, whereas the first hyperpolarizability value is 5.6250 × 10^−30^ esu. A comparative table of the values of the first hyperpolarizability (β) for selected organic NLO cocrystals is presented in Table 2. As stated by Avci et al. and Pandey et al., urea is a basic reference molecule in NLO studies, and is widely used as a benchmark for comparative analysis [66,67]. The calculated value of the first hyperpolarizability of the studied cocrystal is more than 10 times greater than that of urea (β for urea is 0.37289 × 10^−30^ esu), and is comparable to other organic NLO cocrystals, which indicates a potential application of the obtained compound in nonlinear optics.

The calculated average second hyperpolarizability (γ) values of selected organic nonlinear optical materials and the obtained cocrystal are presented in Table 3 and Table S7. The comparison of these values shows that the bis(acridine)–2,4-dihydroxybenzaldehyde cocrystal exhibits a significant γ value of 62.27 × 10^−36^ esu, which is more than three times that of the reference compound *p*-nitroaniline (17.50 × 10^−36^ esu), placing it among the most promising candidates for further exploration in nonlinear optics [78].

## 4. Conclusions

The cocrystalization of acridine with 2,4-dihydroxybenzaldehyde leads to the formation of a 2:1 stoichiometric cocrystal with potential nonlinear optical properties. TG and DSC studies indicate that the obtained compound is stable up to its melting point (141 °C), whereas the thermogram of the physical mixture of reagents confirms the possibility of cocrystal formation upon heating. The obtained compound crystallizes in the noncentrosymmetric *P*2_1_ monoclinic space group, with two acridine and one 2,4-dihydroxybenzaldehyde molecules in the asymmetric unit, and its crystal packing is stabilized by strong O_(aldehyde)_–H⋯N_(acridine)_ and weak C_(aldehyde)_–H⋯O_(aldehyde)_ and C_(aldehyde)_–H⋯O_(aldehyde)_ hydrogen bonds, as well as π_(acridine)_–π_(acridine)_ and C_(acridine)_–H⋯π_(aldehyde)_ interactions. The calculated energy gap (ΔE) between the HOMO and LUMO surfaces (2.434 eV) falls within the expected range for nonlinear optical materials, indicating charge transfer due to π-π* transitions among the molecules. The calculated first (β = 5.63 × 10^−30^ esu) and second hyperpolarizability (γ = 62.27 × 10^−36^ esu) values indicate that the acridine–2,4-dihydroxybenzaldehyde cocrystal is a promising candidate for nonlinear optical applications. The results presented in this study can aid in the rational design of crystals based on supramolecular interactions between aromatic heterocyclic organic compounds and benzaldehydes, leading to the development of materials with nonlinear optical properties.

## Figures and Tables

**Table 1:** Crystal data for bis(ACR)–2,4DHBA cocrystal.

Compound	Bis(ACR)–2,4DHBA
Chemical formula	C_33_H_24_N_2_O_3_
FW/g·mol^−1^	496.55
Crystal system	monoclinic
Space group	*P*2_1_
*a*/Å	7.8366(4)
*b*/Å	17.9803(8)
*c*/Å	8.9765(4)
*α*/°	90
*β*/°	96.347(5)
*γ*/°	90
*V*/Å^3^	1257.09(11)
*Z*	2
*T*/K	291(2)
*λ_Cu_*/Å	1.54184
*ρ_cal_*_c_/g·cm^–3^	1.312
*F*(000)	520
*µ*/mm^−1^	0.675
*θ* range/°	4.92–67.10
Completeness of *θ*/%	97.3
Reflections collected	7275
Reflections unique	3290 [R_int_ = 0.0631]
Data/restraints/parameters	1725/3/351
Goodness of fit on *F*^2^	1.043
Final R_1_ value (*I* > 2σ(*I*))	0.0631
Final *w*R_2_ value (*I* > 2σ(*I*))	0.1206
Final R_1_ value (all data)	0.1312
Final *w*R_2_ value (all data)	0.1610
Largest diff. peak and hole/e·Å^–3^	0.159 and −0.208
CCDC number	2,428,711

**Table 2:** The first hyperpolarizability (β) values of selected NLO cocrystals.

Organic NLO Cocrystals	β (×10^−30^ esu)
chalcone cocrystal [68]	17.30
paracetamol–picric acid cocrystal [69]	4.86
2-amino-5-chloropyridine–3-methylbenzoic acid cocrystal [70]	4.16
5-fluorouracil–salicylic acid cocrystal [71]	2.17
syringic acid–urea cocrystal [72]	0.55
6-mercaptopurine–syringic acid cocrystal [73]	12.88
8-hydroxyquinoline–acetone-(2,4-dinitrophenyl)hydrazone cocrystal [74]	12.47
bis(cinnamic acid)–hexamine cocrystal [75]	5.33
2,3-dimethylquinoxaline–3,5-dinitrobenzoic acid cocrystal [76]	1.27
diisopropylammonium hydrogen squarate cocrystal [77]	4.16
bis(acridine)–2,4-dihydroxybenzaldehyde cocrystal [this work]	5.63

**Table 3:** The average second hyperpolarizability (γ) values of selected organic materials.

Organic NLO Materials	(γ) (×10^−36^ esu)
*p*-nitroaniline [78]	17.50
ferulic acid [79]	16.01
2-(benzylamino)−4-chloro-5-sulfamoylbenzoic acid [80]	33.10
2–bromo-4-nitroaniline [81]	67.96
(E)-4-(3-fluorobenzyloxy)-N’-benzylidenebenzohydrazide [82]	65.63
bis(acridine)-2,4-dihydroxybenzaldehyde cocrystal [this work]	62.27

## Data Availability

The original contributions presented in this study are included in the article. Further inquiries can be directed to the corresponding authors. Crystallographic data for the structure reported in this article have been deposited at the Cambridge Crystallographic Data Centre, under deposition number No. CCDC 2428711. Copies of the data can be obtained free of charge via https://www.ccdc.cam.ac.uk/structures/ (accessed on 4 March 2025).

## Supplementary Materials

The following supporting information can be downloaded at: https://www.mdpi.com/article/10.3390/ma18071492/s1, Figure S1: Crystals of bis(acridine)–2,4-dihydroxybenzaldehyde cocrystal viewed under an optical microscope; Figure S2: Energy framework results for (a) 2,4-dihydroxybenzaldehyde, (b) acridine A, and (c) acridine B molecules, with neighboring molecules generated within a 3.8 Å radius; Table S1: Functional groups and their peaks found in ATR-FTIR spectrum of bis(acridine)–2,4-dihydroxybenzaldehyde; Table S2: Hydrogen bond geometry for title compound; Table S3: Geometry of π–π interactions for title compound; Table S4: C_(acridine)_–H···C_(aromatic)_ interaction geometry for title compound; Table S5: Calculated energy values and corresponding global descriptors; Table S6: The x, y, and z components of the dipole moment (μ), polarizability (α), and first hyperpolarizability; Table S7: The x, y, and z components of average second hyperpolarizability (γ) of acridine-2,4-dihydroxybenzaldehyde cocrystal.
