# Supplementary material for: Synthesis, Structural Characterization, Hirshfeld Surface Analysis, and Evaluation of Nonlinear Optical Properties of Novel Cocrystal of Acridine with 2,4-Dihydroxybenzaldehyde
Source: Materials (Basel). 2025 Mar 27;18(7):1492. doi: 10.3390/ma18071492 (PMC11989604; doi:10.3390/ma18071492)
Supplement: Supplementary file 1 [file materials-18-01492-s001.zip › materials-3538338-supplementary.pdf]

Supporting information

Synthesis, structural characterization, Hirshfeld surface analysis, and evaluation of nonlinear optical properties of novel cocrystal of acridine with 2,4-dihydroxybenzaldehyde

Patryk Nowak ORCID: 0000-0001-6271-6336, Artur Sikorski* ORCID: 0000-0002-4559-7870

University of Gdańsk, Faculty of Chemistry, W. Stwosza 63, 80-308 Gdańsk, Poland

*e-mail: artur.sikorski@ug.edu.pl (A.S.)

Synthesis

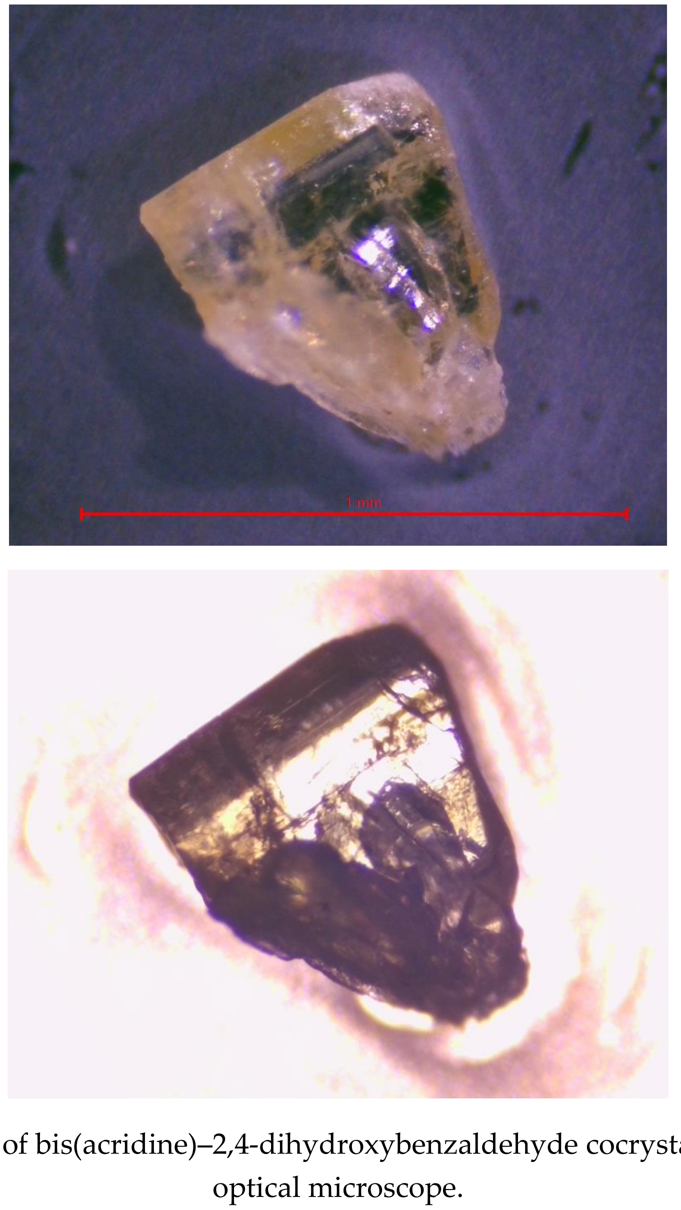

Figure S1. Crystals of bis(acridine)–2,4-dihydroxybenzaldehyde cocrystal viewed under an optical microscope.

Energy framework data

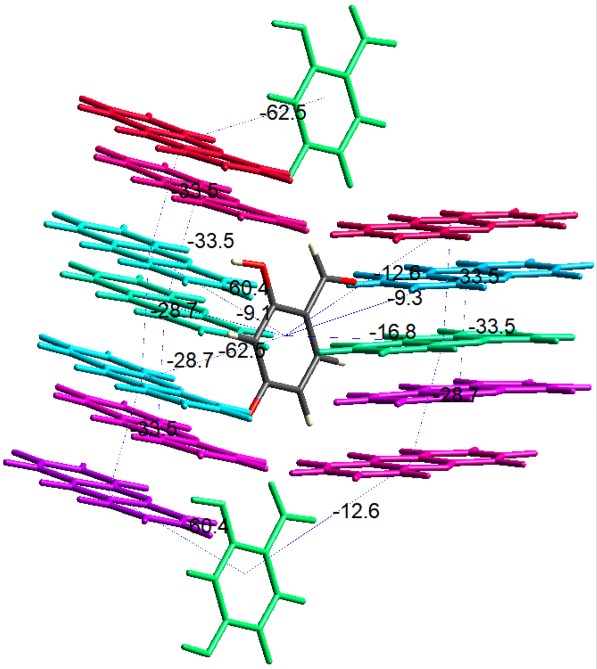

	N	Symop	R	Electron Density	E_ele	E_pol	E_dis	E_rep	E_tot
	0	-	6.03	B3LYP/6-31G(d,p)	-80.3	-21.8	-24.9	97.5	-62.5
	0	-	5.66	B3LYP/6-31G(d,p)	-86.6	-24.0	-25.9	115.7	-60.4
	0	-	4.02	B3LYP/6-31G(d,p)	2.0	-1.5	-47.2	18.5	-28.7
	0	-	6.18	B3LYP/6-31G(d,p)	-3.7	-1.7	-14.1	7.9	-12.6
	0	-	3.87	B3LYP/6-31G(d,p)	-0.8	-2.0	-58.0	31.4	-33.5
	0	-	9.79	B3LYP/6-31G(d,p)	-7.3	-3.2	-5.6	9.1	-9.3
	0	-	7.20	B3LYP/6-31G(d,p)	-2.5	-0.9	-17.0	14.5	-9.1
	0	x, y, z	8.98	B3LYP/6-31G(d,p)	-1.7	-0.3	-8.1	3.0	-7.1
	0	-	9.57	B3LYP/6-31G(d,p)	-0.3	-0.1	-3.5	0.4	-3.2
	0	-x, y+1/2, -z	9.61	B3LYP/6-31G(d,p)	-0.8	-0.1	-2.8	0.4	-3.1
	0	-	8.79	B3LYP/6-31G(d,p)	-2.3	-0.3	-10.0	7.6	-6.8
	0	-	6.41	B3LYP/6-31G(d,p)	-0.5	-0.5	-9.5	3.7	-6.8
	0	-	10.04	B3LYP/6-31G(d,p)	-0.3	-0.2	-6.9	3.5	-4.4
	0	-	10.35	B3LYP/6-31G(d,p)	0.4	-0.1	-3.5	0.6	-2.3
	2	x, y, z	7.84	B3LYP/6-31G(d,p)	-3.0	-1.5	-8.8	14.5	-3.0
	1	-	5.38	B3LYP/6-31G(d,p)	-7.1	-1.6	-21.7	17.6	-16.8
	1	-	7.20	B3LYP/6-31G(d,p)	-2.5	-0.9	-17.0	14.5	-9.1
	1	-	5.66	B3LYP/6-31G(d,p)	-86.6	-24.0	-25.9	115.7	-60.4
	1	-	6.03	B3LYP/6-31G(d,p)	-80.3	-21.8	-24.9	97.5	-62.5
	1	-	9.79	B3LYP/6-31G(d,p)	-7.3	-3.2	-5.6	9.1	-9.3
	0	-	6.96	B3LYP/6-31G(d,p)	-2.4	-1.3	-5.6	0.7	-7.9
	0	-	8.94	B3LYP/6-31G(d,p)	-1.0	-0.7	-5.0	1.3	-5.1
	0	x, y, z	8.98	B3LYP/6-31G(d,p)	-1.7	-0.3	-7.8	2.9	-6.9
	0	-	8.67	B3LYP/6-31G(d,p)	1.9	-0.7	-7.8	4.4	-2.7
	0	-x, y+1/2, -z	9.59	B3LYP/6-31G(d,p)	-0.9	-0.1	-3.1	0.4	-3.5
	0	-	5.38	B3LYP/6-31G(d,p)	-7.1	-1.6	-21.7	17.6	-16.8
	0	-	7.22	B3LYP/6-31G(d,p)	0.6	-0.3	-9.2	4.0	-5.2
	0	-	8.95	B3LYP/6-31G(d,p)	-3.1	-0.5	-1.8	0.0	-5.2
	1	-	8.95	B3LYP/6-31G(d,p)	-3.1	-0.5	-1.8	0.0	-5.2
	1	-	8.67	B3LYP/6-31G(d,p)	1.9	-0.7	-7.8	4.4	-2.7
	1	-	7.22	B3LYP/6-31G(d,p)	0.6	-0.3	-9.2	4.0	-5.2
	1	-	6.41	B3LYP/6-31G(d,p)	-0.5	-0.5	-9.5	3.7	-6.8
	1	-	8.94	B3LYP/6-31G(d,p)	-1.0	-0.7	-5.0	1.3	-5.1
	1	-	6.18	B3LYP/6-31G(d,p)	-3.7	-1.7	-14.1	7.9	-12.6
	1	-	6.96	B3LYP/6-31G(d,p)	-2.4	-1.3	-5.6	0.7	-7.9

(a)

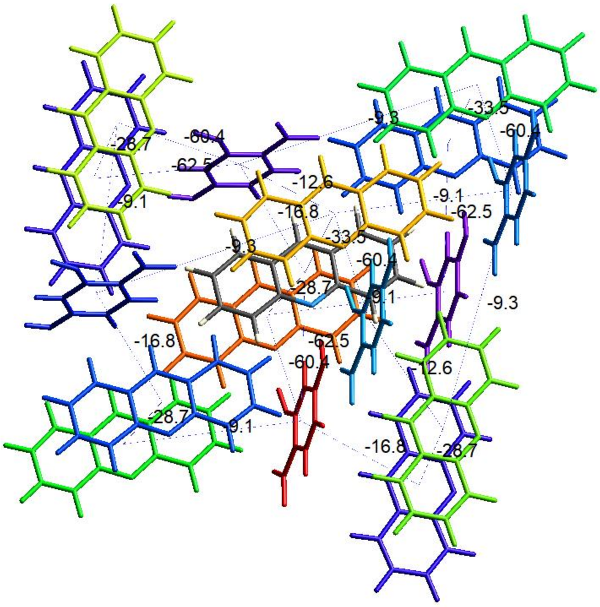

	N	Symop	R	Electron Density	E_ele	E_pol	E_dis	E_rep	E_tot
	1	-	6.03	B3LYP/6-31G(d,p)	-80.3	-21.8	-24.9	97.5	-62.5
	0	-	5.66	B3LYP/6-31G(d,p)	-86.6	-24.0	-25.9	115.7	-60.4
	1	-	4.02	B3LYP/6-31G(d,p)	2.0	-1.5	-47.2	18.5	-28.7
	0	-	6.18	B3LYP/6-31G(d,p)	-3.7	-1.7	-14.1	7.9	-12.6
	1	-	3.87	B3LYP/6-31G(d,p)	-0.8	-2.0	-58.0	31.4	-33.5
	0	-	9.79	B3LYP/6-31G(d,p)	-7.3	-3.2	-5.6	9.1	-9.3
	0	-	7.20	B3LYP/6-31G(d,p)	-2.5	-0.9	-17.0	14.5	-9.1
	0	x, y, z	8.98	B3LYP/6-31G(d,p)	-1.7	-0.3	-8.1	3.0	-7.1
	1	-	9.57	B3LYP/6-31G(d,p)	-0.3	-0.1	-3.5	0.4	-3.2
	0	-x, y+1/2, -z	9.61	B3LYP/6-31G(d,p)	-0.8	-0.1	-2.8	0.4	-3.1
	1	-	8.79	B3LYP/6-31G(d,p)	-2.3	-0.3	-10.0	7.6	-6.8
	0	-	6.41	B3LYP/6-31G(d,p)	-0.5	-0.5	-9.5	3.7	-6.8
	1	-	10.04	B3LYP/6-31G(d,p)	-0.3	-0.2	-6.9	3.5	-4.4
	1	-	10.35	B3LYP/6-31G(d,p)	0.4	-0.1	-3.5	0.6	-2.3
	0	x, y, z	7.84	B3LYP/6-31G(d,p)	-3.0	-1.5	-8.8	14.5	-3.0
	0	-	5.38	B3LYP/6-31G(d,p)	-7.1	-1.6	-21.7	17.6	-16.8
	0	-	7.20	B3LYP/6-31G(d,p)	-2.5	-0.9	-17.0	14.5	-9.1
	0	-	5.66	B3LYP/6-31G(d,p)	-86.6	-24.0	-25.9	115.7	-60.4
	0	-	6.03	B3LYP/6-31G(d,p)	-80.3	-21.8	-24.9	97.5	-62.5
	0	-	9.79	B3LYP/6-31G(d,p)	-7.3	-3.2	-5.6	9.1	-9.3
	1	-	6.96	B3LYP/6-31G(d,p)	-2.4	-1.3	-5.6	0.7	-7.9
	1	-	8.94	B3LYP/6-31G(d,p)	-1.0	-0.7	-5.0	1.3	-5.1
	2	x, y, z	8.98	B3LYP/6-31G(d,p)	-1.7	-0.3	-7.8	2.9	-6.9
	1	-	8.67	B3LYP/6-31G(d,p)	1.9	-0.7	-7.8	4.4	-2.7
	2	-x, y+1/2, -z	9.59	B3LYP/6-31G(d,p)	-0.9	-0.1	-3.1	0.4	-3.5
	1	-	5.38	B3LYP/6-31G(d,p)	-7.1	-1.6	-21.7	17.6	-16.8
	1	-	7.22	B3LYP/6-31G(d,p)	0.6	-0.3	-9.2	4.0	-5.2
	0	-	8.95	B3LYP/6-31G(d,p)	-3.1	-0.5	-1.8	0.0	-5.2
	0	-	8.95	B3LYP/6-31G(d,p)	-3.1	-0.5	-1.8	0.0	-5.2
	0	-	8.67	B3LYP/6-31G(d,p)	1.9	-0.7	-7.8	4.4	-2.7
	0	-	7.22	B3LYP/6-31G(d,p)	0.6	-0.3	-9.2	4.0	-5.2
	0	-	6.41	B3LYP/6-31G(d,p)	-0.5	-0.5	-9.5	3.7	-6.8
	0	-	8.94	B3LYP/6-31G(d,p)	-1.0	-0.7	-5.0	1.3	-5.1
	0	-	6.18	B3LYP/6-31G(d,p)	-3.7	-1.7	-14.1	7.9	-12.6
	0	-	6.96	B3LYP/6-31G(d,p)	-2.4	-1.3	-5.6	0.7	-7.9

(b)

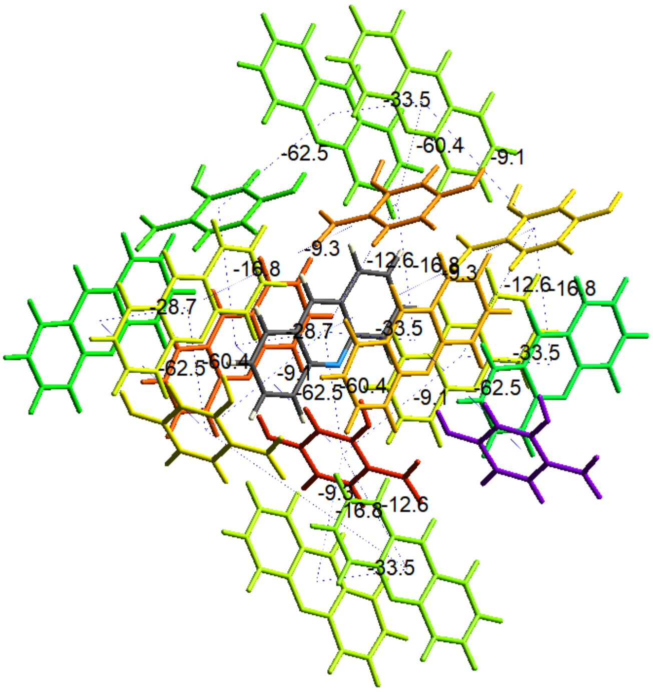

	N	Symop	R	Electron Density	E_ele	E_pol	E_dis	E_rep	E_tot
	0	-	6.03	B3LYP/6-31G(d,p)	-80.3	-21.8	-24.9	97.5	-62.5
	1	-	5.66	B3LYP/6-31G(d,p)	-86.6	-24.0	-25.9	115.7	-60.4
	1	-	4.02	B3LYP/6-31G(d,p)	2.0	-1.5	-47.2	18.5	-28.7
	1	-	6.18	B3LYP/6-31G(d,p)	-3.7	-1.7	-14.1	7.9	-12.6
	1	-	3.87	B3LYP/6-31G(d,p)	-0.8	-2.0	-58.0	31.4	-33.5
	1	-	9.79	B3LYP/6-31G(d,p)	-7.3	-3.2	-5.6	9.1	-9.3
	1	-	7.20	B3LYP/6-31G(d,p)	-2.5	-0.9	-17.0	14.5	-9.1
	2	x, y, z	8.98	B3LYP/6-31G(d,p)	-1.7	-0.3	-8.1	3.0	-7.1
	1	-	9.57	B3LYP/6-31G(d,p)	-0.3	-0.1	-3.5	0.4	-3.2
	2	-x, y+1/2, -z	9.61	B3LYP/6-31G(d,p)	-0.8	-0.1	-2.8	0.4	-3.1
	1	-	8.79	B3LYP/6-31G(d,p)	-2.3	-0.3	-10.0	7.6	-6.8
	1	-	6.41	B3LYP/6-31G(d,p)	-0.5	-0.5	-9.5	3.7	-6.8
	1	-	10.04	B3LYP/6-31G(d,p)	-0.3	-0.2	-6.9	3.5	-4.4
	1	-	10.35	B3LYP/6-31G(d,p)	0.4	-0.1	-3.5	0.6	-2.3
	0	x, y, z	7.84	B3LYP/6-31G(d,p)	-3.0	-1.5	-8.8	14.5	-3.0
	0	-	5.38	B3LYP/6-31G(d,p)	-7.1	-1.6	-21.7	17.6	-16.8
	0	-	7.20	B3LYP/6-31G(d,p)	-2.5	-0.9	-17.0	14.5	-9.1
	0	-	5.66	B3LYP/6-31G(d,p)	-86.6	-24.0	-25.9	115.7	-60.4
	0	-	6.03	B3LYP/6-31G(d,p)	-80.3	-21.8	-24.9	97.5	-62.5
	0	-	9.79	B3LYP/6-31G(d,p)	-7.3	-3.2	-5.6	9.1	-9.3
	0	-	6.96	B3LYP/6-31G(d,p)	-2.4	-1.3	-5.6	0.7	-7.9
	0	-	8.94	B3LYP/6-31G(d,p)	-1.0	-0.7	-5.0	1.3	-5.1
	0	x, y, z	8.98	B3LYP/6-31G(d,p)	-1.7	-0.3	-7.8	2.9	-6.9
	0	-	8.67	B3LYP/6-31G(d,p)	1.9	-0.7	-7.8	4.4	-2.7
	0	-x, y+1/2, -z	9.59	B3LYP/6-31G(d,p)	-0.9	-0.1	-3.1	0.4	-3.5
	0	-	5.38	B3LYP/6-31G(d,p)	-7.1	-1.6	-21.7	17.6	-16.8
	0	-	7.22	B3LYP/6-31G(d,p)	0.6	-0.3	-9.2	4.0	-5.2
	1	-	8.95	B3LYP/6-31G(d,p)	-3.1	-0.5	-1.8	0.0	-5.2
	0	-	8.95	B3LYP/6-31G(d,p)	-3.1	-0.5	-1.8	0.0	-5.2
	0	-	8.67	B3LYP/6-31G(d,p)	1.9	-0.7	-7.8	4.4	-2.7
	0	-	7.22	B3LYP/6-31G(d,p)	0.6	-0.3	-9.2	4.0	-5.2
	0	-	6.41	B3LYP/6-31G(d,p)	-0.5	-0.5	-9.5	3.7	-6.8
	0	-	8.94	B3LYP/6-31G(d,p)	-1.0	-0.7	-5.0	1.3	-5.1
	0	-	6.18	B3LYP/6-31G(d,p)	-3.7	-1.7	-14.1	7.9	-12.6
	0	-	6.96	B3LYP/6-31G(d,p)	-2.4	-1.3	-5.6	0.7	-7.9

(c)

Figure S2. Energy framework results for (a) 2,4-dihydroxybenzaldehyde, (b) acridine A, and (c) acridine B molecules, with neighboring molecules generated within a 3.8 Å radius.

ATR-FTIR data

Table S1. Functional groups and their peaks found in ATR-FTIR spectrum of bis(acridine)–2,4-dihydroxybenzaldehyde.

Absorption Peaks (cm^{-1})	Functional Groups
3054-2500	Aromatic C–H (stretching)
1655	Carbonyl C=O
1619–1404	Skeletal C=C and C=N
1263, 1222	Phenolic C-O
724	Aromatic C-H (bending)

Crystallographic data

Table S2. Hydrogen bonds geometry for title compound.

D–H···A	*d*(D–H) [Å]	*d*(H···A) [Å]	*d*(D···A) [Å]	∠D–H···A (°)
O29–H29···N10B[i]	1.00(12)	1.70(12)	2.68(8)	167(13)
O30–H30···N10A[i]	1.00(7)	1.75(7)	2.74(8)	171(4)
C3B–H3B···O28[ii]	0.93	2.48	3.23(12)	138
C27–H27···O30[iii]	0.93	2.44	3.37(11)	177
Symmetry code: (i) x,y,z; (ii) 2-x,1/2+y,-z; (iii) 1+x,y,z				

Table S3. π–π stacking interactions geometry for title compound.

CgI[a]	CgJ[a]	CgI···CgJ[b] [Å]	Dihedral angle[c] [°]	Interplanar distance[d] [Å]	Offset[e] [Å]
Cg(1)	Cg(7)[i]	3.848(5)	3.4(4)	3.446(3)	1.594
Cg(1)	Cg(7)[ii]	4.043(5)	3.4(4)	3.720(3)	1.578
Cg(1)	Cg(8)[i]	3.695(5)	2.8(4)	3.456(3)	1.241
Cg(1)	Cg(9)[ii]	3.802(5)	2.5(4)	3.720(3)	0.832
Cg(2)	Cg(7)[ii]	3.788(5)	4.3(4)	3.694(4)	0.781
Cg(2)	Cg(8)[i]	3.834(5)	3.5(4)	3.451(4)	1.577
Cg(2)	Cg(8)[ii]	4.033(5)	3.5(4)	3.691(4)	1.621
Cg(3)	Cg(7)[i]	3.790(5)	3.0(4)	3.482(4)	1.465
Cg(3)	Cg(9)[i]	3.916(5)	2.7(5)	3.495(4)	1.599
Cg(3)	Cg(9)[ii]	4.001(5)	2.7(5)	3.656(4)	1.484
Cg(7)	Cg(1)[ii]	4.043(5)	3.4(4)	3.722(3)	1.584
Cg(7)	Cg(1)[iii]	3.848(5)	3.4(4)	3.502(3)	1.711
Cg(7)	Cg(2)[ii]	3.788(5)	4.3(4)	3.707(3)	0.838
Cg(7)	Cg(3)[iii]	3.791(5)	3.0(4)	3.496(3)	1.498
Cg(8)	Cg(1)[iii]	3.696(5)	2.8(4)	3.481(4)	1.308
Cg(8)	Cg(2)[ii]	4.033(5)	3.5(4)	3.693(4)	1.627
Cg(8)	Cg(2 [iii]	3.834(5)	3.5(4)	3.495(4)	1.670
Cg(9)	Cg(1)[ii]	3.801(5)	2.5(4)	3.709(4)	0.780
Cg(9)	Cg(3)[ii]	4.001(5)	2.7(5)	3.715(4)	1.624
Cg(9)	Cg(3)[iii]	3.916(5)	2.7(5)	3.575(4)	1.767
Symmetry code: (i) -1+x,y,z; (ii) x,y,z; (iii) 1+x,y,z					

(a) Cg represents the centre of gravity of the rings. (b) Cg···Cg is the distance between ring centroids. (c) The dihedral angle is that between the mean planes of Cg(I) on ring J. (d) The interplanar distance is the perpendicular distance from CgI to ring J. (e) The offset is the perpendicular distance of CgJ on ring I.

Table S4. $C_{(acridine)}$–H···$C_{(aromatic)}$ interaction geometry for title compound.

**C–H···A**	***d*(H···A) [Å]**	***d*(C···A) [Å]**	**∠C–H···A [°]**
C1A–H1A···C23	2.97	3.91	171
C9A–H9A···C26	2.76	3.65	162
C6B–H6B···C26	2.76	3.64	158
C7B–H7B···C23	3.25	4.10	149

HOMO-LUMO data

Table S5. Calculated energy values and corresponding global descriptors.

**Molecular properties**	ACR-24DHBA
Total energy (Hartree)	-1606.681
HOMO (eV)	-5.417
LUMO (eV)	-2.983
ΔE (eV)$_{(HOMO-LUMO)}$	2.434
Ionization potential (eV)	5.417
Electron affinity (eV)	2.983
Dipole moment (Debye)	16.381

NLO data

The x, y, and z components of the dipole moment (μ), polarizability (α), and first hyperpolarizability (β) were derived from the Gaussian output file and calculated using the following equations:

$$\mu_{tot} = \sqrt{\{\mu_x^2 + \mu_y^2 + \mu_z^2\}}$$

$$\alpha_{tot} = \frac{1}{3}(\alpha_{xx} + \alpha_{yy} + \alpha_{zz})$$

$$\Delta\alpha = \frac{1}{\sqrt{2}}\sqrt{(\alpha_{xx} - \alpha_{yy})^2 + (\alpha_{yy} - \alpha_{zz})^2 + (\alpha_{zz} - \alpha_{xx})^2 + 6\alpha_{xz}^2 + 6\alpha_{xy}^2 + 6\alpha_{yz}^2}$$

$$\beta_{tot} = \sqrt{(\beta_{xxx} + \beta_{xyy} + \beta_{xzz})^2 + (\beta_{yyy} + \beta_{yzz} + \beta_{yxx})^2 + (\beta_{zzz} + \beta_{zxx} + \beta_{zyy})^2}$$

Table S6. The x, y, and z components of the dipole moment (μ), polarizability (α), and first hyperpolarizability.

**Component**	**Value (Debye)**	**Component**	**Value (× 10 $^{-23}$ esu)**	**Component**	**Value (× 10 $^{-30}$ esu)**
μ_x	15.5845	α_{xx}	6.3884	β_{xxx}	2.4965
μ_y	5.0344	α_{xy}	-0.6044	β_{xxy}	-2.4386
μ_z	0.3291	α_{yy}	6.3593	β_{xyy}	-0.0530
$\boldsymbol{\mu_{tot}}$	**16.381**	α_{xz}	-0.4495	β_{yyy}	-0.4079
		α_{yz}	0.9761	β_{xxz}	3.5134
		α_{zz}	4.0881	β_{xyz}	-1.5963
		$\boldsymbol{\alpha_{tot}}$	**5.6119 × 10^{-23}**	β_{yyz}	-0.4444
		**Δα**	**3.1282 × 10^{-23}**	β_{xzz}	1.0355
				β_{yzz}	-0.3935
				β_{zzz}	-0.0625
				$\boldsymbol{\beta_{tot}}$	**5.6250 × 10^{-30}**

The x, y, and z components of the average second hyperpolarizability ($\langle\gamma\rangle$) was derived from the Gaussian output file and calculated using the following equation:

$$\langle\boldsymbol{\gamma}\rangle = \frac{\mathbf{1}}{\mathbf{5}}\left(\boldsymbol{\gamma}_{xxxx} + \boldsymbol{\gamma}_{yyyy} + \boldsymbol{\gamma}_{zzzz} + \mathbf{2}\left(\boldsymbol{\gamma}_{xxyy} + \boldsymbol{\gamma}_{xxzz} + \boldsymbol{\gamma}_{yyzz}\right)\right)$$

Table S7. The x, y, and z components of average second hyperpolarizability $\langle\boldsymbol{\gamma}\rangle$ of acridine-2,4-dihydroxybenzaldehyde cocrystal.

Component	Value ($\times10^{-35}$)
γ_{xxxx}	5.584089
γ_{yyyy}	5.117841
γ_{zzzz}	7.165512
γ_{xxyy}	1.892258
γ_{xxzz}	2.56572
γ_{yyzz}	2.294438
